# WellInverter: a web application for the analysis of fluorescent reporter gene data

**DOI:** 10.1186/s12859-019-2920-4

**Published:** 2019-06-11

**Authors:** Yannick Martin, Michel Page, Christophe Blanchet, Hidde de Jong

**Affiliations:** 1 Univ. Grenoble Alpes, Inria, Grenoble, 38000 France; 2 Univ. Grenoble Alpes, Grenoble IAE, Grenoble, 38000 France; 3 Institut Français de Bioinformatique, IFB-core, Orsay, 91403 France

**Keywords:** Fluorescent reporter gene data, Microplate reader, Quantitative modeling of gene expression, Estimation, Web application

## Abstract

**Background:**

Fluorescent reporter genes have become widely used for monitoring gene expression in living cells. When a microbial strain carrying a reporter gene is grown in a microplate reader, the fluorescence and the absorbance (optical density) of the culture can be automatically measured every few minutes in a highly parallelized way. The extraction of useful information from the resulting large amounts of data is not easy to achieve, because the fluorescence and absorbance measurements are only indirectly related to promoter activities and protein concentrations, requiring mathematical models of the expression of reporter genes for their interpretation. Although the principles of the analysis of reporter gene data are well-established today, there is a lack of general-purpose bioinformatics tools based on generic measurement models and sound inference procedures. This has motivated the development of WellInverter, a web application based on well-known methods for regularized linear inversion.

**Results:**

We present a new version of WellInverter that considerably improves the performance and usability of the original application. In particular, we have put in place a parallel computing architecture with a load balancer to distribute analysis queries over several back-end servers, we have completely redesigned the graphical user interface to better support the different analysis steps, and we have developed a plug-in system for the parsing of data files produced by microplate readers from different manufacturers. We illustrate the functioning of WellInverter by analyzing data of the expression of a fluorescent reporter gene controlled by a phage promoter in growing *Escherichia coli* populations. We show that the expression pattern in different growth media, supporting different growth rates, corresponds to the pattern expected for a constitutive gene.

**Conclusions:**

The new version of WellInverter is a robust, easy-to-use and scalable web application, which has been deployed on two publicly accessible web servers and which can also be installed locally. A demo version of the application with two sample datasets is available on-line.

**Electronic supplementary material:**

The online version of this article (10.1186/s12859-019-2920-4) contains supplementary material, which is available to authorized users.

## Background

**Fig. 1:**
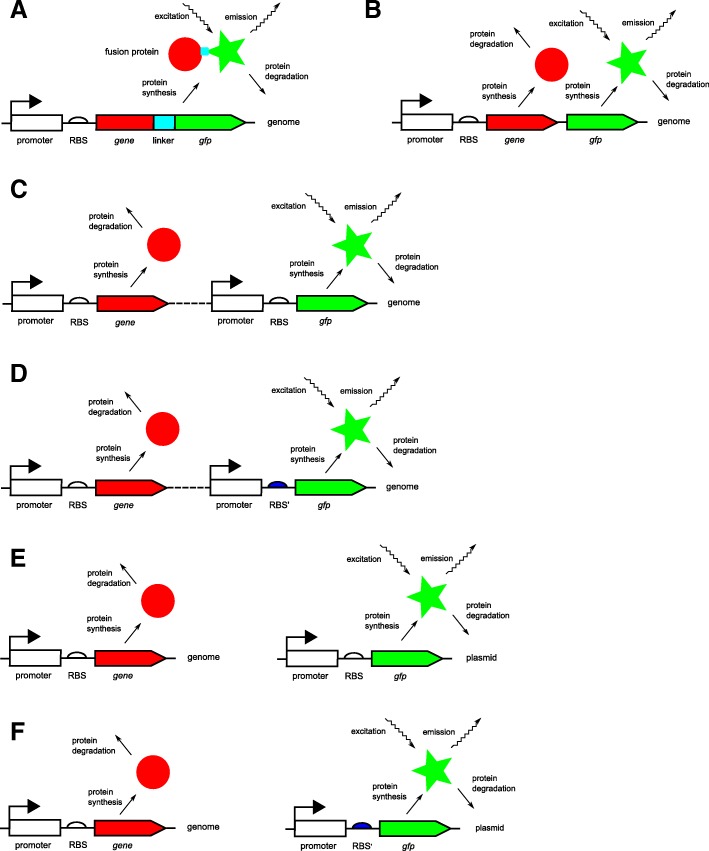
Relation between the measurements of fluorescence and absorbance in reporter gene experiments and the activity of the host gene and the concentration of the protein it encodes. **a**: Co-expression of the host and reporter gene, together encoding a single fusion protein. **b**: Idem, but the host gene and reporter gene code for two distinct proteins. **c**: Co-regulation of the host and reporter genes, which share the same transcriptional and translational regulatory sequences (promoter and ribosome binding site, RBS). **d**: Idem, but only transcriptional regulation is shared between the host and reporter genes as indicated by the different colors of the RBSs. **e-f**: As in **c**-**d**, but the reporter gene is carried on a plasmid rather than in the chromosome. In order to avoid visual clutter, the transcription and translation processes in the figure have been collapsed into a single protein synthesis step

Fluorescent reporter genes are powerful tools for measuring gene expression in individual cells or in populations of cells. A fluorescent reporter gene codes for a protein that has a characteristic fluorescence emission spectrum when excited with light at a specific wavelength [1, 2]. The principle of the use of fluorescent reporters for quantifying gene expression is based on cloning the reporter gene downstream of a gene or a regulatory region of interest, either in the genome or on a plasmid that can be transformed into the host cell [3]. By construction, the reporter protein is co-expressed with the protein of interest (when the reporter gene and the gene of interest code for a single fusion protein) or the expression of the two proteins is co-regulated (when the two genes share transcriptional and/or translational regulatory regions). The intensity of light emitted by a cell or a population of cells provides an indication of the quantity of fluorescent protein present and therefore reports on the quantity of the protein of interest or on its synthesis rate (Fig. 1).

Contrary to genome-wide methods for measuring gene expression, like RNA-Seq and quantitative proteomics [4, 5], the use of fluorescent reporters allows only one to three genes per cell to be monitored in parallel. However, gene expression can be quantified in vivo, without the need to harvest and lyse the cells, and at high sampling density, typically once every few minutes. Over the years, many useful resources have appeared, such as libraries of reporter strains for model organisms [3] and computer tools for designing reporter plasmids [6]. Moreover, the widespread adoption of thermostated microplate readers in experimental laboratories has made it possible to automate and multiplex reporter gene assays on the population level. This has resulted in large time-series data sets, typically comprising 10^5^−10^6^ measurements for 10^2^ wells on the microplate. The analysis of these data sets has shed new light on the functioning of complex regulatory networks [7–11].

The primary data obtained from reporter gene experiments in microplate readers consist of measured intensities of the fluorescence emitted by a growing population of cells as well as absorbance (optical density) measurements reflecting the size of the population. Due to the indirect relation of these measurements with the expression of the gene of interest (Fig. 1), the interpretation of the primary data is not straightforward. Therefore, the inference of the activity of a gene or the concentration of a protein from fluorescence and absorbance measurements has become a lively research area over the past decade. Different methods have been proposed, all based on dynamical models that describe the process of gene expression, using different procedures to infer quantities of interest [10, 12–23].

In previous work we have shown how the interpretation of reporter gene data can be formulated as an input estimation problem and solved in a mathematically sound and practically useful manner using linear inversion [22]. Most existing approaches first smooth the primary data and then inject the resulting approximating functions into the gene expression model, which may lead to estimation errors that are difficult to control. Instead of this indirect approach, we formulated a global optimization problem with a regularization term directly applying to the quantities to be estimated. This problem was solved using efficient linear inversion methods [24–26] adapted to the structure of the problem. The methods have been implemented in the Python package WellFARE and made accessible through the web application WellInverter. WellInverter allows the user to upload, analyze, and visualize the data of a reporter gene experiment as well as downloading the results for further processing. The analysis includes outlier detection, background correction, estimation of growth rate, promoter activity, and protein concentration, the computation of summary statistics over the wells of the microplate, and the export of the analysis results.

The previous version of WellInverter [22] had a number of shortcomings that limited its practical usability for a broad audience of biologists and bioinformaticians. First, since the estimation of growth rates, promoter activities, and protein concentrations may be computationally costly, especially when several wells are analyzed in parallel and when the application has to simultaneously handle several queries coming from different users, response times could become prohibitively long. Second, the user interface was functional but rudimentary, and did not support important features of the WellFARE algorithms and different background correction procedures. Third, the application was limited to the import of data files from one commercially-available microplate reader and support for other file formats would have required the development of new, customized parsers.

The new version of WellInverter presented in this paper addresses all of the above problems. In particular, we have put in place a parallel computing architecture with a load balancer to distribute the estimation queries over several back-end servers, we have completely redesigned the graphical user interface to better support the different analysis steps, and we have developed a plug-in system for the parsing of data files produced by microplate readers from different manufacturers. This has resulted in a scalable and user-friendly web service providing a guaranteed quality of service in terms of availability and response time. To our knowledge, this is the first easily accessible and broadly applicable web application for the analysis of reporter gene experiments. The WellInverter web server has been deployed on the cloud of the French Institute of Bioinformatics (IFB) and on the servers of our host institute, but is also available as a stand-alone version that can be locally installed (see *Availability and requirements* section).

In order to illustrate the use of WellInverter, we apply it to the analysis of reporter gene experiments in the enterobacterium *Escherichia coli* that quantify the expression of a reporter gene controlled by a constitutive promoter. Transcription from a constitutive promoter is not regulated by specific transcription factors and thus depends only on the activity of the transcriptional machinery, that is, the concentration of (free) RNA polymerase and precursor pools [27, 28]. Constitutive promoters are important as experimental controls when estimating the effect of transcriptional regulators on gene expression, as they capture the global effect of changes in cell physiology independently from the specific effect of transcription factors [7, 11]. We provide further evidence that the promoter considered here, the phage promoter pRM [29], is truly constitutive by comparing the steady-state expression levels of the reporter protein with a theoretical model of gene expression from constitutive promoters [28].

## Implementation

### Estimation algorithms

The estimation of promoter activities and protein concentrations from measured fluorescence intensities and absorbance (optical density) values follows the approach developed previously [22], which we briefly summarize here. Expression of the fluorescent reporter gene is modeled by the ordinary differential equation model

1 $$ \frac{d}{dt} R(t) = a(t) \cdot V(t) - \gamma_{r}\cdot R(t),   $$

where *R*(*t*) [mmol] is the time-varying quantity of the fluorescent reporter protein in the growing bacterial population, *a*(*t*) [mmol min ^−1^ L ^−1^] the synthesis rate of the reporter protein per unit of population volume *V*(*t*) [L], and *γ*_*r*_ the degradation constant of the reporter [min ^−1^]. The protein synthesis rate is often called gene activity or promoter activity in the reporter gene literature [10], motivated by the fact that, under certain assumptions and as a first approximation, the rates of transcription and translation are proportional [11]. The degradation constant is related to the half-life *t*_1/2_ of the protein by *t*_1/2_= ln2/*γ*_*r*_, which can be easily measured [17].

The absorbance and fluorescence measurements carried out in microplate readers are assumed to be proportional to the volume of the growing bacterial population and the total reporter protein quantity in the population, respectively. More precisely, the absorbance and fluorescence measurements $\tilde {V}, \tilde {R}$ at time-point *t*_*i*_ are related to the volume and reporter protein quantities by means of the following measurement model

2 $$\begin{array}{*{20}l} \tilde{V}(t_{i}) & = \alpha\, V(t_{i}) + \nu_{i}, \end{array} $$

3 $$\begin{array}{*{20}l} \tilde{R}(t_{i}) & = \beta\, R(t_{i}) + \nu^{\prime}_{i}, \end{array} $$

where $\nu _{i}, \nu ^{\prime }_{i}$ represent measurement noise. The proportionality constants *α*,*β* are usually unknown, without the performance of nontrivial calibration experiments. It immediately follows from the above definitions that the concentration of the reporter protein in the growing population at time-point *t*_*i*_ is proportional to $\tilde {R}(t_{i})/\tilde {V}(t_{i})$.

A first estimation problem consists in inferring the growth rate of the population from the absorbance measurements *via* the following growth equation

4 $$ \frac{d}{dt} (\alpha \, V)(t) = \alpha \, V(t) \, \mu(t) \approx \bar{V}(t)\, \mu(t),   $$

where *μ*(*t*) [min ^−1^] represents the growth rate and $\bar {V}(t)$ an interpolated version of the measurements $\tilde {V}(t)$. With the latter approximation, the population volume *α* *V*(*t*) is the output of a linear system with input *μ*(*t*) and initial conditions (*α* *V*)(*t*_0_). The estimation of the growth rate and the initial population volume from the observed output $\tilde {V}$ at the time-points *t*_*i*_ is an instance of a so-called linear inversion problem that can be efficiently solved using standard methods [24–26]. In order to make the problem well-posed, we assume that the input *μ*(*t*) is piecewise-constant and subject to an appropriate regularization condition [22]. In particular, we impose Tikhonov regularization on the first derivative of the growth rate [30], penalizing rapid successive variations, and we set the regularization parameter by generalized cross validation [31].

Solving the above estimation problem yields, as a side product, a denoised estimate $\widehat {\alpha V}$ of the population volume. This estimate can be used for a second estimation problem, namely to infer *a*(*t*), the promoter activity, from the absorbance and fluorescence measurements *via* Eq. 1. We rewrite the latter as

5 $$ \frac{d}{dt} R(t) = \frac{a(t)}{\alpha} \cdot \widehat{\alpha V}(t) - \gamma_{r}\cdot R(t),   $$

and note that this yields again a linear, time-varying system with input *a*(*t*) and output *R*(*t*). The input can be estimated, up to a multiplicative constant due to the fact that *α*,*β* are not known, by means of the same linear inversion approach as above [22].

A third estimation problem concerns the inference of the concentration of the protein of interest from the absorbance and fluorescence data. We make the assumption that the reporter gene and the host gene are co-regulated, giving rise to the same promoter activity *a*(*t*), but allow the reporter and host proteins to have different degradation constants (corresponding to cases **b**-**e** in Fig. 1). In other words, we combine Eq. 5 with the following model of host protein expression:

6 $$\begin{array}{*{20}l} \frac{d}{dt} P(t) & = \frac{a(t)}{\alpha} \cdot \widehat{\alpha V}(t) - \gamma_{p}\cdot P(t),  \end{array} $$

7 $$\begin{array}{*{20}l} p(t) & = \alpha \cdot P(t)/ \widehat{\alpha V}(t),  \end{array} $$

where *P*(*t*) [mmol] is the time-varying quantity of the host protein, *p*(*t*) [mmol L ^−1^] its concentration, and *γ*_*p*_ [h ^−1^] its degradation constant. We assume *γ*_*p*_ to be approximately known, bearing in mind that most bacterial proteins are stable, with half-lives well over 10 h [32]. If the reporter protein is stable as well, the default choice of *γ*_*p*_=*γ*_*r*_ usually leads to good results, in the sense of returning a protein concentration that is a smoothed version of the reporter concentration. The system of Eqs. 5-7 is again linear, where the equations for the reporter and host protein quantity are coupled by a shared input *a*(*t*). The linear inversion procedure can be adapted for linear equations with a shared input, resulting in an estimate of *p*(*t*) from absorbance and fluorescence measurements, given values for *γ*_*p*_,*γ*_*r*_ [22].

The linear inversion procedures for estimating the growth rate, promoter activity, and protein concentration from population-level absorbance and fluorescence data have been implemented in the Python package WellFARE [22]. A new version correcting several (minor) bugs and some new functionalities has been released since the original publication (see “Availability and requirements” section).

### Distributed computing architecture

The WellFARE package has been integrated into the web application WellInverter, thus allowing non-expert users to analyze their data in a user-friendly way without installing the package and developing Python scripts. WellInverter has a client-server architecture: the client can access the application through a web browser and the estimation queries are handled by a dedicated server running WellFARE.

The estimation procedures may be computationally complex, because the algorithms involve numerical simulation of the models and the multiplication and inversion of large matrices [22]. The complexity is determined by the number of so-called control points, a high number of control points corresponding to a high temporal resolution of the estimates of promoter activities and protein concentrations. The user can set the number of control points in WellInverter. For long time-series (> 200 measurements) with a large number of control points (> 180), the computation of the promoter activity for all wells of a microplate may take a few minutes. This led to scalability problems in the original WellInverter architecture when the estimation procedures needed to be repeated over several wells or when several users launched estimation procedures in parallel.

**Fig. 2:**
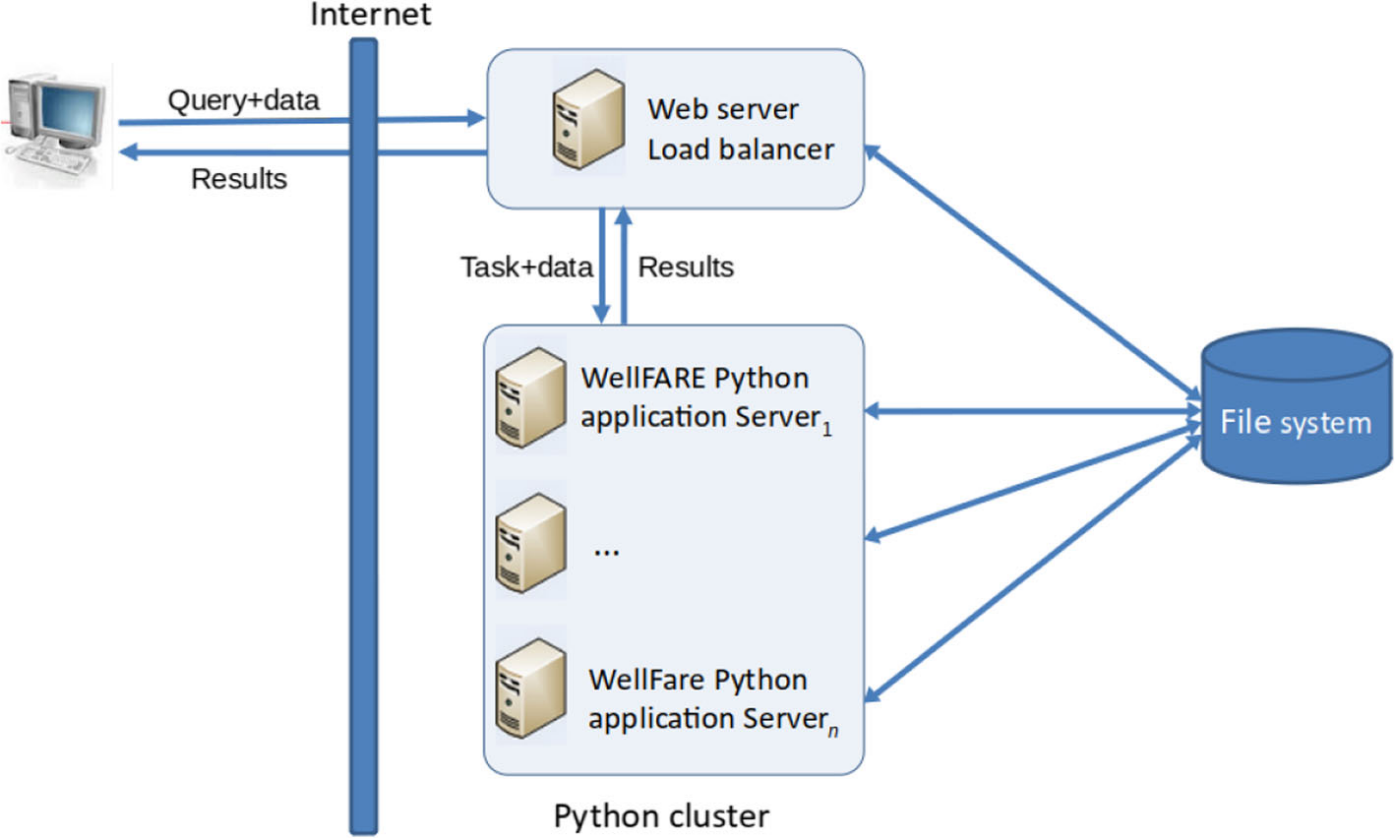
Distributed architecture of WellInverter. User queries are received by a front-end web server that distributes them over several back-end Python application servers running WellFARE, using the load balancer. The front-end server is also responsible for user authentification and logging traces of user operations. The results are sent back to the user for display in a web browser

Scalability problem are frequently encountered in interactive web applications and also in high-traffic web sites. The usual solution to this problem is an architecture consisting of a cluster of servers rather than a single server, enabling the parallelization of query handling. There are other advantages of this distributed architecture, such as a higher fault tolerance. Figure 2 shows the improved WellInverter architecture, in which several back-end Python application servers execute time-consuming computational tasks as well as user/data management tasks, including the (incremental) saving of analysis results. A front-end web server receives user queries and distributes these over the Python servers, using a so-called load balancer.

We developed a general-purpose Python load balancer, pyLoadBalancer, which is also available independently of WellInverter (see “Availability and requirements” section). As the computations for determining growth rates, promoter activities, and protein concentrations are completely independent for each well, deploying the estimation procedures over a computer cluster makes it possible to considerably reduce the computation time. In the design of the architecture, care has been taken to ensure data security and safety, in particular by providing an appropriate authentication layer and log files.

We carried out computational experiments to quantify the performance of the parallel architecture. When calculating the promoter activity for 55 outlier-filtered and background-corrected wells in the microplate experiment attached as Additional file 1, we found that by increasing the number of servers from 1 to 8, the execution time decreases three-fold (from 20.3 s to 6.9 s). In addition to improving the speed of execution of a single job, the architecture also allows several jobs to be executed in parallel. The redesign of WellInverter has thus led to a distributed architecture that is scalable, robust, and safe.

### Graphical user interface

The client part of WellInverter consists of the graphical user interface (GUI) of the application, running in a web browser. The GUI of the original WellInverter was functional but limited, in the sense that it did not support the entire workflow and the whole range of options of the WellFARE algorithms. We therefore designed and developed a completely new GUI, written in JavaScript and communicating with the server using JSON-encoded data and Ajax (Asynchronous JavaScript) calls [33, 34]. The GUI is structured around a visual representation of the microplate and guides the user through the process of analyzing reporter gene data in an intuitive manner, as will be illustrated in some detail in the “Results” section. The user can switch between different screens that provide different types of operations on the wells of the microplate.

In the *Outlier filtering* screen, the user has the choice between manual and automatic filtering of outliers. Outliers may arise from occasional instrument failure or from the use of beads in the wells to improve oxygen transfer in the culture. The outliers are caused by the deflection of light by the glass bead when the latter finds itself in the path of the light beam at the time of the measurement. During manual outlier filtering, the user can either select individual data points as outliers or sketch a trend line with a tolerance band such that all data points outside the tolerance band are considered outliers. Automatic outlier filtering relies on the iterative application of the Savitsky-Golay smoothing algorithm as implemented in SciPy [35]. The user can specify the different parameters of this algorithm, including the size of the smoothing window, a cut-off parameter defined in terms of the standard deviation of the distance of the data points to the smoothed curve, and the number of iterations of the filtering procedure. Automatic outlier filtering can be launched for a single well or for a subsets of the wells on the plate. The results of the outlier filtering procedure can be visually displayed, by selecting a well in the plate or by hovering the mouse button over a well. In order to visually assess the results, outlier data points can be included or left out from the plot.

The *Background correction* screen aims at subtracting the background of the measured fluorescence and absorbance time-courses. This is a critical step for the interpretation of the results of a reporter gene experiment and several solutions have been proposed for achieving this [17, 21, 36]. In WellInverter, the user has the choice between three different background correction procedures. First, a user-defined baseline can be substracted from the measured absorbance and fluorescence values. This solution is appropriate if the background level does not change with the growth of the bacterial culture, as is for example the case for the absorbance background and for the autofluorescence of bacteria in some regions of the emission spectrum (e.g., red in the case of *E. coli*). If the autofluorescence does vary with the population size, as is for example the case for green autofluorescence, a second solution consists in directly subtracting the fluorescence emitted by a culture with bacteria carrying a promoter-less reporter plasmid or (which in many cases comes to the same thing) bacteria without the reporter plasmid. This solution works well if the strains with and without an (active) reporter plasmid have the same growth kinetics. The GUI of WellInverter allows the user to define background wells for different sections of the microplate, in accordance with the design of the experiment, by a few mouse clicks.

If the strains with and without an (active) reporter plasmid do not have the same growth kinetics, a third option can be chosen that is based on the construction of a so-called calibration curve [11]. The calibration curve returns the autofluorescence levels in the control strain as a function of the absorbance levels. Background correction then consists in subtracting the autofluorescence level corresponding to the absorbance level observed for the strain of interest carrying the reporter plasmid (see Fig. 5 below). Care should be taken, however, that the growth kinetics for the two strains are not too dissimilar, as this may reflect serious differences in the growth physiology of the two strains. The results of the background correction step, for individual wells, are shown in the *Outlier filtering* screen. The possibility to construct a calibration curve from the data, by defining the upper and lower bounds, the smoothing window, and an extrapolation interval, was not offered in the previous version of WellInverter.

The *Plots* screen allows the users to visually inspect the primary and corrected data, as well as the different quantities computed from the data by means of the WellFARE algorithms. In the case of absorbance data, the growth rate can be estimated and displayed. In the case of fluorescence data, the promoter activity, the reporter concentration, and the protein concentration can be estimated and displayed. The user can choose the color of the plotted time-courses and zoom in on specific portions of the time course. For a given well, several quantities can be shown simultaneously, for example the absorbance of a growing bacterial culture and the promoter activity. This makes it possible to visually relate changes in the one to changes in the other. Moreover, the quantities for several wells can be displayed simultaneously, by selecting the wells by means of the mouse. For each of the selected wells, all relevant information can be shown, but it is also possible to limit the displayed results to summary statistics, such as the mean and an uncertainty band defined by the standard deviation or the standard error at each time-point.

In order to facilitate the inspection of several wells in parallel, the new version of WellInverter allows wells to be grouped together in so-called well groups, corresponding to the different conditions in the experimental design. Typical well groups consist of all replicates of the growth of a specific reporter strain in a specific medium. If a well is included in a well group, selecting this well causes all other wells in the group to be selected as well, thus speeding up the inspection of the results of the experiment.

In the *Parameters* screen, the default parameter values for the model, for the background substraction procedures, and for the inference method can be set. In particular, for the model the values of the degradation constants are needed and the growth rate can be declared to be positive. For the inference algorithms, the regularization parameters can be specified and the number of control points chosen, contrary to the previous version of WellInverter. The different parameters for the automatic outlier correction procedure can be chosen as well as a fixed absorbance and fluorescence background level for background subtraction.

### Plug-in system to parse data files

A major practical challenge for programs supporting the analysis of reporter gene data is that different microplate reader manufacturers use different formats for the output data files generated during the experiment. These files need to be parsed in order to fill the WellInverter data structures with the primary data from the instrument. In the absence of a standard file format, this requires a specific parser to be developed for each microplate reader or for each output file format for a given microplate reader. The development of a robust parser, however, requires specific programming skills that many potential WellInverter users do not possess.

**Fig. 3:**
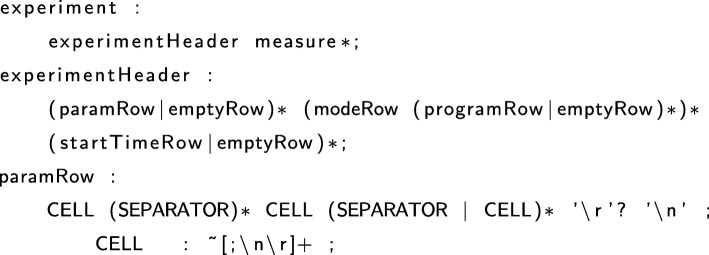
Excerpt of the grammar for defining input data file formats, here for parsing data files produced by a Tecan microplate reader. The grammar, specified in EBNF format [39], defines the overall structure of the file and consists of a header describing the experiment (experimentHeader) followed by zero, one or several measurement blocks (measure). experimentHeader is defined in the second expression and paramRow, one of the elements of experimentHeader, is further specified in the third definition. The full grammar definition is supplied with the stand-alone version of the software

In order to work around this complication, the new version of WellInverter employs a so-called grammar-based parser generator, which supports the high-level specification of data formats and their automatic conversion into working parsers [37]. We have chosen ANTLR [38], which allows the data format to be formulated as a grammar from which a parser is generated that is capable of interpreting files specified in the language defined by the grammar. In ANTLR, grammars are specified in the EBNF formalism [39]. An excerpt of the grammar for parsing data files produced by a Tecan microplate reader is shown in Fig. 3. When defining a grammar for a new data file format, it is usually convenient to start from existing grammars that have been developed already and that come with WellInverter.

The grammar specification is transformed by ANTLR into Java code, the parser, which can be called by WellInverter when an experiment data file is uploaded. The parsers are plug-ins of WellInverter, declared on the server side of the application, thus making their development, addition, and maintenance entirely modular. A few parser file are available by default, notably for Tecan and Perkin-Elmer microplate readers, and new parser plug-ins will be added as they are developed.

### Data export

The GUI offers general file management operations for loading and saving the results of (the analysis of) a reporter gene experiment. For many purposes, it is desirable to export the data in formats that allow them to be analyzed by other software, such as general purpose scientific programming languages like Matlab, Python, and R. In the new version of WellInverter, the contents of the *Plots* screen can be exported in CSV and XLS format, in addition to several graphical formats (PDF, JPEG, PNG, SVG). Moreover, the user can export the data and/or the final or intermediate analysis results in the JSON format, a format that is widely used in the scientific computing community. The precise contents of the JSON export file can be customized in the *Export* screen. The file structure is defined in the WellInverter manual. The JSON file defined in the *Export* screen is different from the JSON file produced when saving an experiment in the general file management menu. The latter file is used for archival purposes and, while containing all information for reproducing the analysis results (definition of outliers, background wells, and well groups), it does not contain the analysis results themselves.

## Results

### Reporter gene experiments with a constitutive phage promoter in *E. coli*

As a typical WellInverter use case, we will discuss the analysis of reporter gene experiments carried out with a transcriptional fusion of a constitutive promoter and a *gfp* reporter gene in *E. coli*. By definition, constitutive promoters are not regulated by any transcription factor, so that transcription initiated from a constitutive promoter only depends on the activity of the gene expression machinery, that is, the concentrations of (free) RNA polymerase and precursor pools [27, 28]. Changes in the activity of the gene expression machinery reflect changes in the global cell physiology. The latter occur due to a change in the environment, such as the gradual depletion of the growth substrate, and they affect the expression of all genes of the bacterium. This makes constitutive promoters a useful control for assessing the regulatory effect of transcription factors on a target promoter. In particular, the specific effect of a transcription factor should subsist after correcting for the effect of the global cell physiology captured by the activity profile of the constitutive promoter [7, 28]. As an alternative to constitutive promoters, measurements of the growth rate have been used as a read-out of the global cell physiology [8].

It is not easy to ascertain that a promoter is constitutive, as this requires establishing a negative result, the absence of an effect from (known and unknown) transcription factors of the bacterial cell. Usually, promoters are considered constitutive if there exist *a-priori* arguments that they are not subject to regulation by host factors and if their activity profiles closely resemble those of known constitutive promoters. For instance, the pRM promoter of phage *λ* [29] used in this study is not a native *E. coli* promoter and the known regulators of pRM in *λ* phage can be assumed absent in *E. coli* cells not infected by the phage. Moreover, the activity profile of the pRM promoter was found similar to that of the well-known synthetic promoter ptet [7].

There also exists a more theoretical approach for testing the constitutiveness of a promoter. Klumpp et al. [28] developed a model predicting how the steady-state concentration of a reporter protein expressed from a constitutive promoter in *E. coli* varies with the growth rate. The predictions of this model were found to agree quite well with the observed concentrations of the LacZ reporter expressed from known constitutive promoters. In this study we will carry out this additional test for the pRM promoter, by computing the growth-rate dependency of the steady-state GFP concentration from reporter gene data and by comparing the observed relation with the theoretically-predicted curve.

The wild-type *E. coli* strain used in the reporter gene experiments described below is a derivative of the BW25113 strain [40]. The wild-type strain was transformed with a pUA66 reporter plasmid carrying a transcriptional fusion of the pRM promoter region and the *gfp* sequence encoding the fast-folding and long-lived GFPmut2 reporter, as described previously [11]. The folding time of GFPmut2 is on the order of a few minutes [3], while its half-life is almost 20 h [7]. The pUA66 reporter plasmid is a low-copy plasmid that is kanamycin-resistant and has the pSC101 origin of replication [3].

Wild-type strains without and with the reporter plasmid, henceforth referred to as WT and WT pRM-*gfp*, respectively, were recovered from glycerol stock (-80 ^o^C) and grown overnight (about 16 h) at 37 ^o^C, with shaking at 200 rpm, in M9 minimal medium [41] supplemented with 0.2% D-glucose and mineral trace elements. Kanamycin (50 mg/ml) was added in the case of WT pRM-*gfp*. The overnight cultures were diluted to an OD_600_ of 0.02 into a 96-well microplate. The wells of the microplate contained 150 *μ*l of M9 minimal medium or LB medium supplemented with different carbon sources (D-glucose, sodium acetate, glycerol, D-galactose, D-maltose) at a final concentration of 0.1% as well as a sterile 2-mm glass bead to improve aeration. No antibiotics were added at this stage. A transparent cover was put on the plates to avoid evaporation, and the microplate cultures were then grown at 37 ^o^C in a microplate reader (Tecan Infinite 200 PRO). The absorbance (600 nm) and the fluorescence (485/535 nm) were read every 2 min, preceded by a 30-s stirring step (orbital and linear shaking, 5 mm amplitude).

In the remainder of this section, we analyze the results of the above reporter gene experiments using WellInverter.

### Analysis of reporter gene data

Access to WellInverter is gained by either typing the server address in the web browser or running a local installation of the application. The file produced by the microplate reader is then imported *via* the *New experiment* option in the *Experiment* menu, in this case using the plug-in for Tecan files with data in row format. This uploads the primary data into the application, including the experimenal settings of the microplate reader stored in the data file. If the experiment has been imported already, the data and the actual state of the analysis can be accessed *via* the *Open experiment* option.

**Fig. 4:**
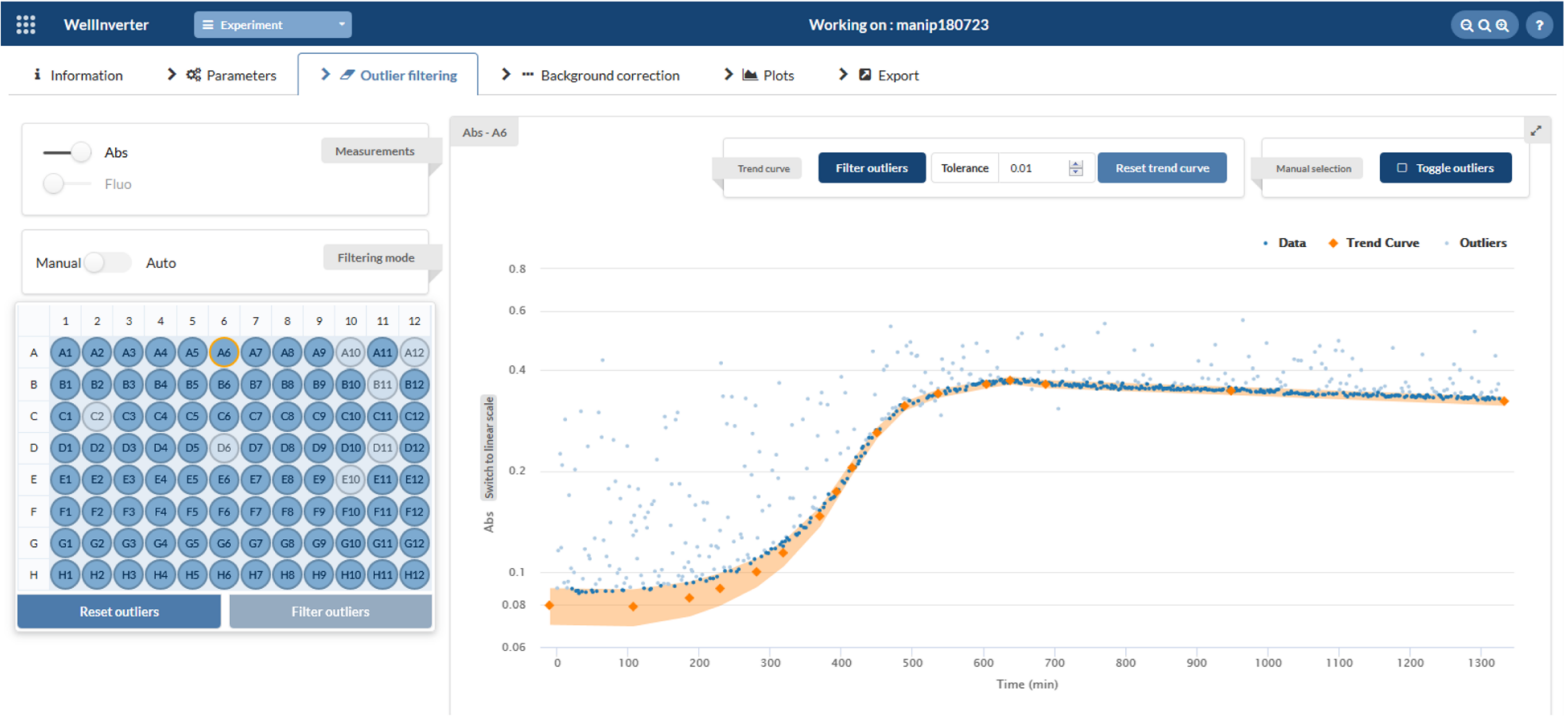
Primary data of a reporter gene experiment. The plot in the WellInverter interface shows (in logarithmic scale) the fluorescence and absorbance measurements in well A6 of the microplate, containing a WT pRM-*gfp* culture growing in M9 minimal medium with 0.1% glucose. Outliers have been filtered using the trend curve defined by the orange band

Figure 4 shows the WellInverter plot of a typical time-course measurement of absorbance and fluorescence in a microplate well containing a WT pRM-*gfp* culture growing in M9 minimal medium with 0.1% glucose. The measurements have been corrected for outliers due to the use of glass beads by means of the manual outlier identification functionality of WellReader. At around 500 min, a break in the growth curve occurs because all glucose in the medium has been consumed. Growth continues at a lower rate until about 650 min, utilizing the acetate that was produced by overflow metabolism during growth on glucose [42].

**Fig. 5:**
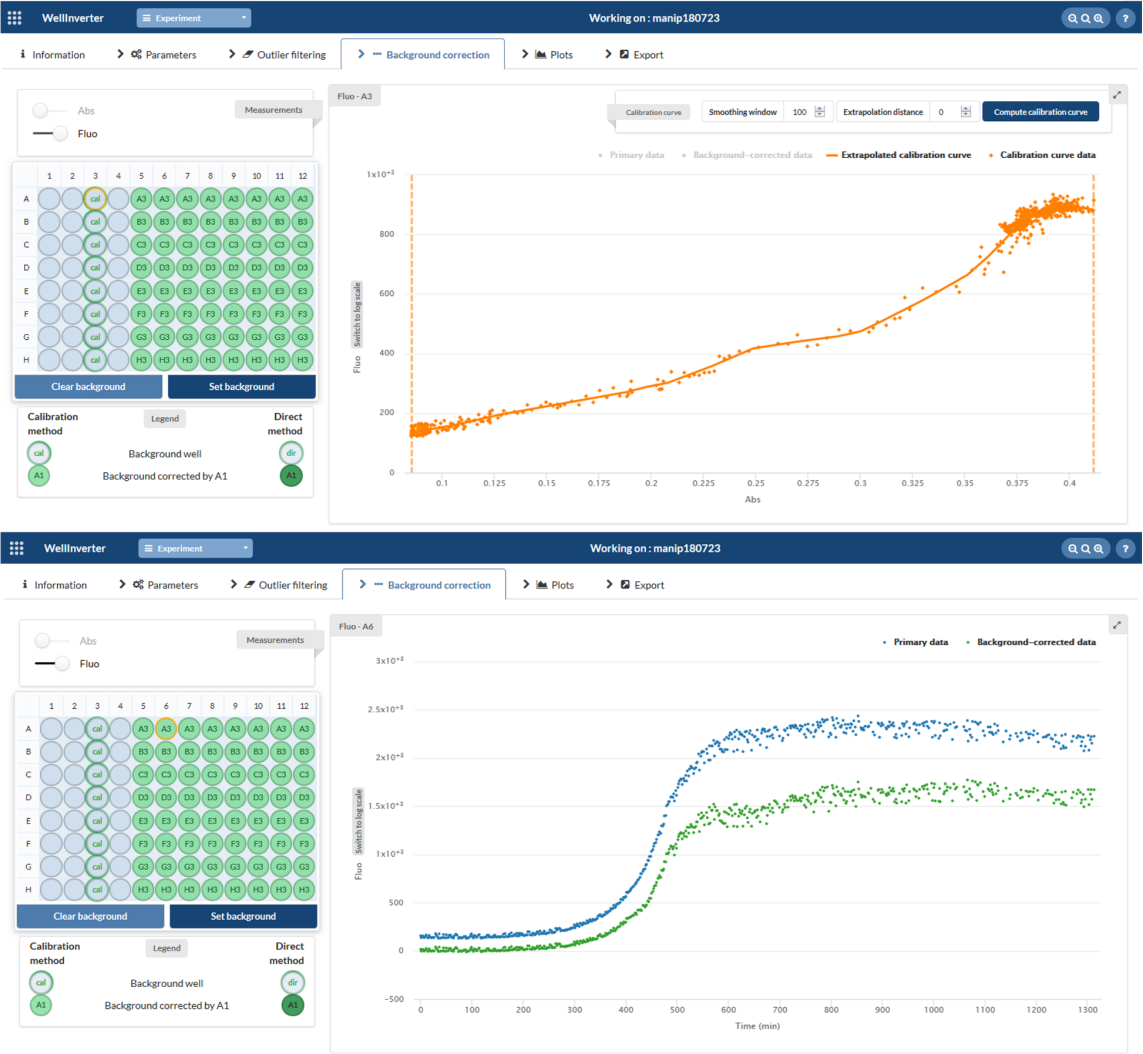
Substraction of background fluorescence. **Upper:** Calibration curve constructed from the fluorescence and absorbance data in well A3, defined as the background for wells A5-A12. The calibration curve is a smoothing spline with a user-defined smoothing parameter. The curve can be extrapolated outside the range of observed absorbance values, a feature that is not needed in this experiment. **Lower:** The primary fluorescence data (blue) and the data after background substraction (green) for well A6

The data in Fig. 4 need to be corrected for background absorbance and fluorescence. We correct the absorbance by subtracting a fixed background value, the observed mean of the absorbance over all M9 minimal medium wells on the plate. This value (0.084) is entered into the *Default background value* field of the *Parameters* menu. As described in the “Implementation” section, the fluorescence background can be corrected in several different ways. We here use calibration curves, which generally lead to robust results. The fluorescence emitted by the WT culture is used for the construction of the calibration curve [11]. In the example at hand, we construct a calibration curve for each of the different growth conditions (media compositions), where each condition corresponds to a row of the microplate. In the case of M9 minimal medium with 0.1% glucose, corresponding to the first row, the background well for the WT pRM-*gfp* cultures growing in wells A5-A12 is chosen to be A3. Figure 5 shows the calibration curve relating the absorbance to the fluorescence, obtained by fitting a smoothing spline to the data. The original fluorescence signal (blue) and the background-corrected signal (green) for well A5 are also shown. As expected, the background-corrected fluorescence signal starts from values close to 0, reflecting the fact that the population density of the *E. coli* cells in the beginning of the experiment, after dilution of the overnight preculture, is very low and that the fluorescence emitted by the cells at this stage is consequently negligible.

**Fig. 6:**
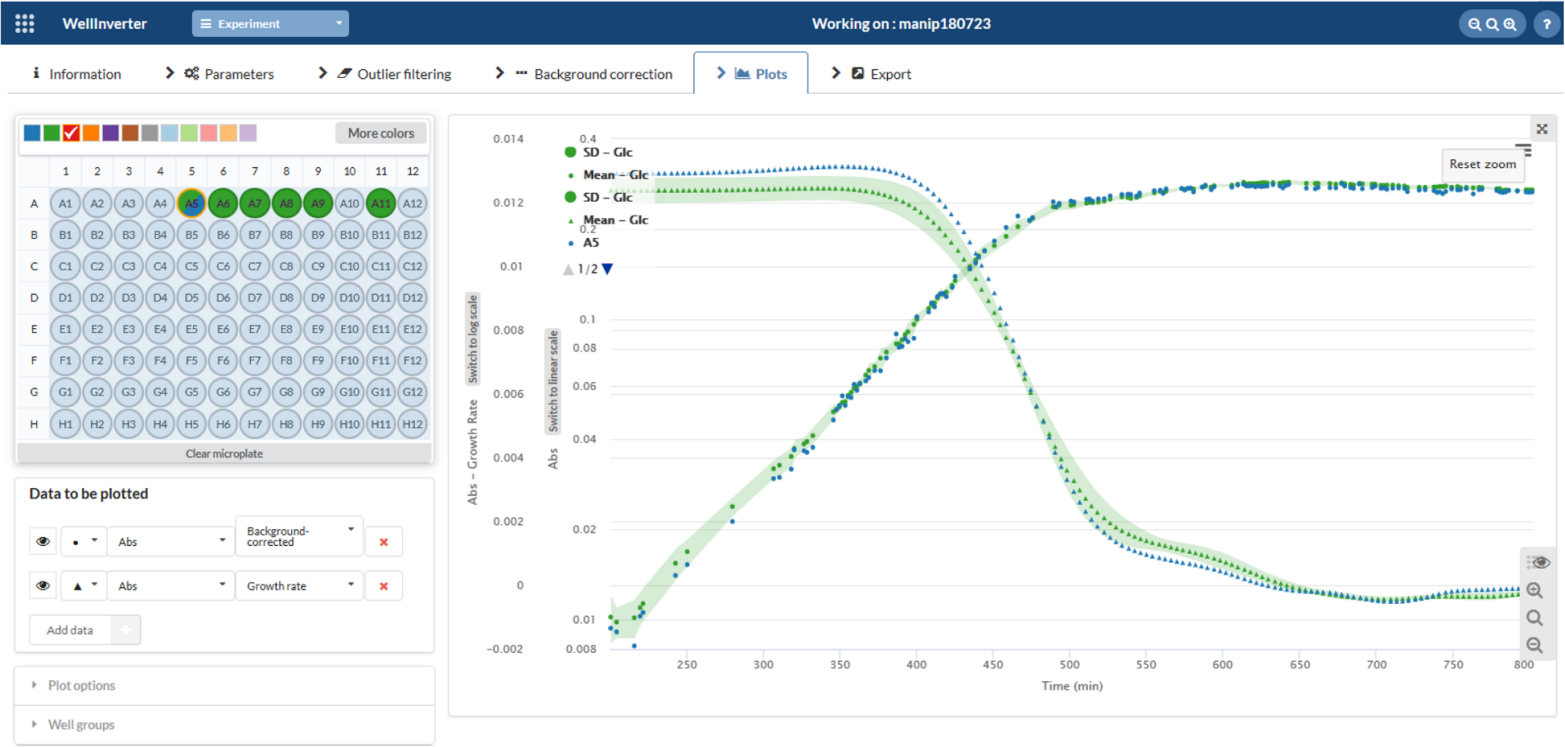
Computation of growth rate. Mean absorbance and growth rate curves and an uncertainty band defined by the standard deviation for a group of wells containing WT pRM-*gfp* cultures growing in M9 minimal medium with 0.1% glucose (green). In addition, the plot shows the time-varying absorbance and growth rate for one sample well in the group (A5, blue). The plot zooms in on the interval [200, 800]

The growth rates of the WT pRM-*gfp* cultures are computed in the *Plots* window. Figure 6 shows the background-corrected absorbance (in logarithmic scale) and the growth rate for a group of wells containing M9 minimal medium with 0.1% glucose. The mean (green dots) and the standard deviation (green band) are plotted, in the interval between 200 and 800 min, as well as the background-corrected absorbance and the growth rate for one well (A5, blue). The observed growth rate during exponential growth in M9 minimal medium with glucose is around 0.0125 min ^−1^, corresponding to a doubling time of around 55 min, as expected in these conditions [43]. Absorbance values that are well below the background absorbance level (0.084) are not reliable and come with large uncertainty bands, so we do not compute growth rates here. For absorbance values higher than 0.1 the growth starts to decline, probably due to oxygen transfer limitations at high bacterial densities. After glucose exhaustion around 500 min the growth rate drops towards 0. The computation of the growth rate needs a regularization parameter that can be set in the *Parameters* window. Here we choose a value in the range that was shown in previous work, by means of simulation studies, to lead to good results [22]. As a visual check of the effect of regularization, WellInverter also allows the absorbance predicted by the model for the estimated growth rate to be plotted and compared with the measured absorbance values.

**Fig. 7:**
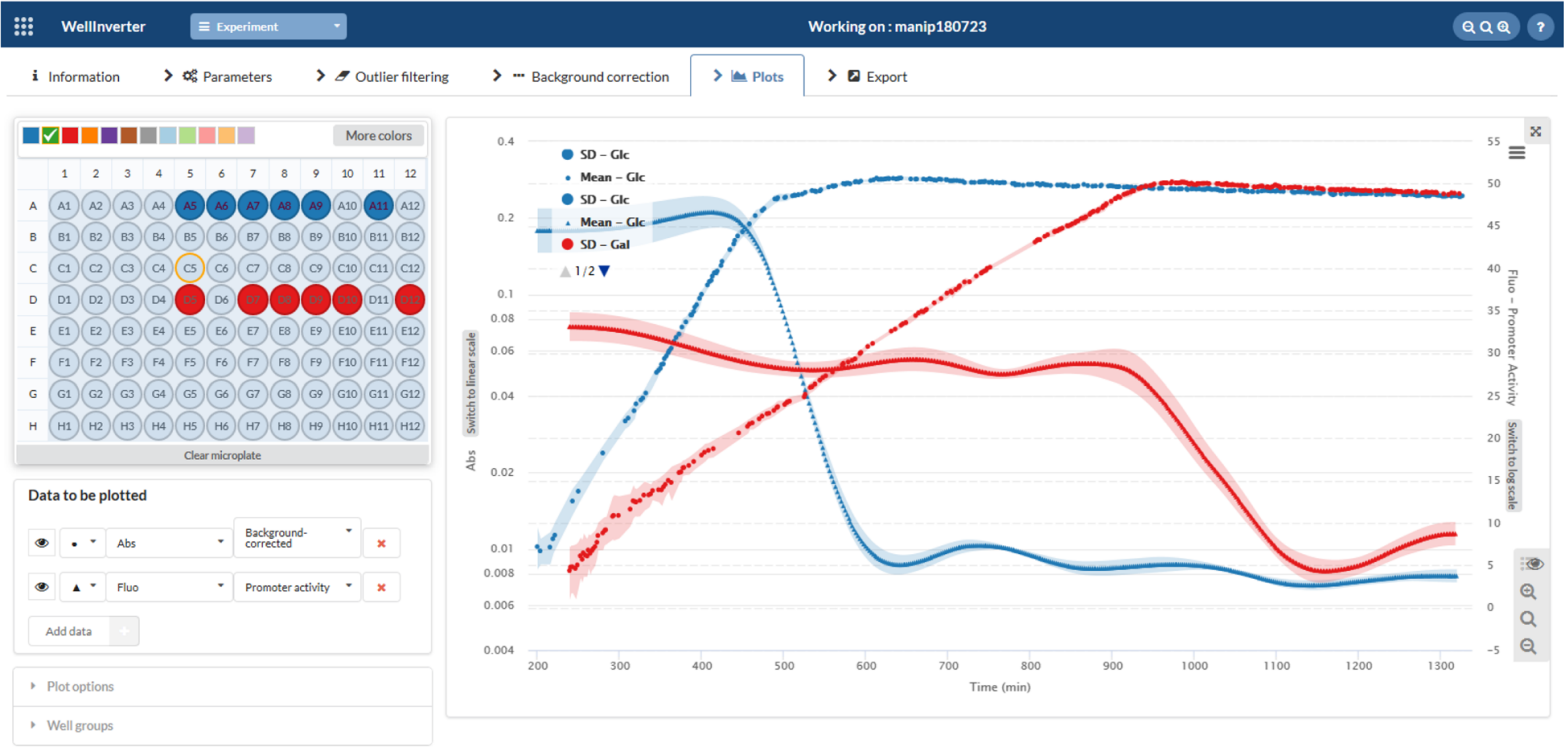
Computation of promoter activities. Mean absorbance and promoter activity curves and an uncertainty band defined by the standard deviation for groups of wells containing WT pRM-*gfp* cultures growing in either M9 minimal medium with 0.1% glucose (blue) or 0.1% galactose (red)

The activity of the pRM promoter in the different WT pRM-*gfp* cultures can also be computed in the *Plots* window. Figure 7 shows the background-corrected absorbance (in logarithmic scale) and pRM activity for a group of wells containing M9 minimal medium with 0.1% glucose and another group of wells containing M9 minimal medium with 0.1% galactose. Both the mean and standard deviation are plotted, along with the background-corrected absorbance. The promoter activities start to drop at the end of exponential growth on glucose and settle at a low level after glucose exhaustion. Note that both the growth rate and the promoter activity are lower during growth on galactose than on glucose (the growth rate is given by the slope of the absorbance curve in logarithmic scale). The lower promoter activity is probably due to the lower activity of the gene expression machinery, for example the lower concentration of free RNA polymerase, at a lower growth rate [44].

**Fig. 8:**
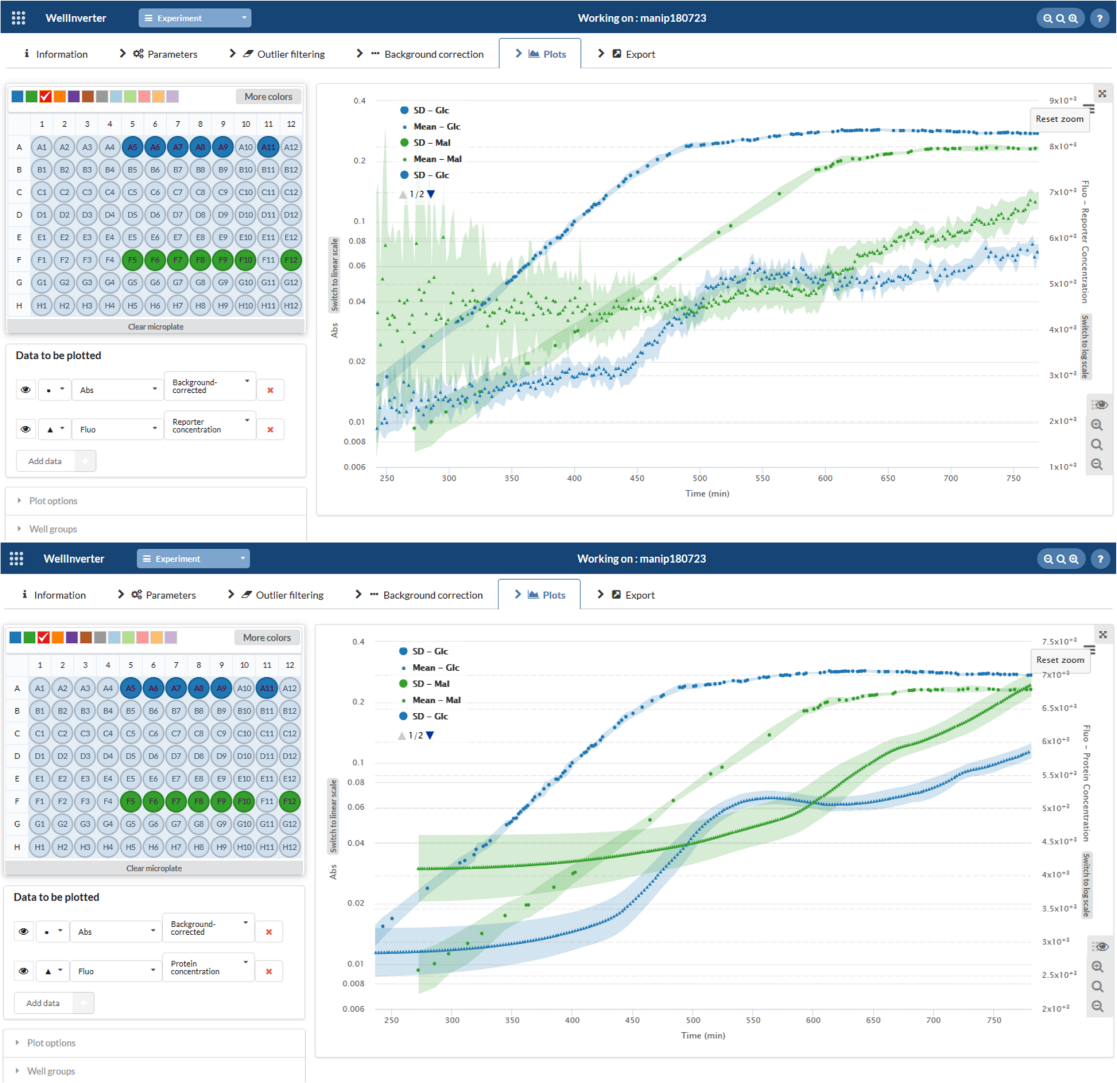
Computation of reporter concentrations. Mean absorbance and reporter concentration curves and an uncertainty band defined by the standard deviation for a group of wells containing WT pRM-*gfp* cultures growing in M9 minimal medium with 0.1% glucose (blue) or 0.1% maltose (green). **Upper:** Reporter concentration estimates obtained by dividing fluorescence by absorbance. **Lower:** Smoothed estimates of reporter concentrations obtained by computing the protein concentration while setting the degradation constant of the host protein equal to the degradation constant of the GFP reporter in the *Parameters* window (0.00065 min ^−1^). Notice that the two estimates are not shown on the same scale

As explained in the section on the Estimation algorithms, the reporter protein concentration can be estimated by dividing the (background-corrected) fluorescence by the (background-corrected) absorbance. Moreover, a smoothed estimate of the reporter concentration can be obtained by computing the protein concentration from the fluorescence data while assuming that the reporter and the host protein have the same half-life. Figure 8 shows the results thus obtained for the WT pRM-*gfp* cultures growing in M9 minimal medium with 0.1% glucose or in M9 minimal medium with 0.1% galactose. In both situations, we see that the estimated reporter concentration is steady during exponential growth on the carbon source and increases upon growth arrest. The reporter concentration is lower in the conditions with faster growth, expressing that the higher promoter activity of the constitutive promoter is outweigthed by the higher rate at which the reporter protein is diluted out [28].

**Fig. 9:**
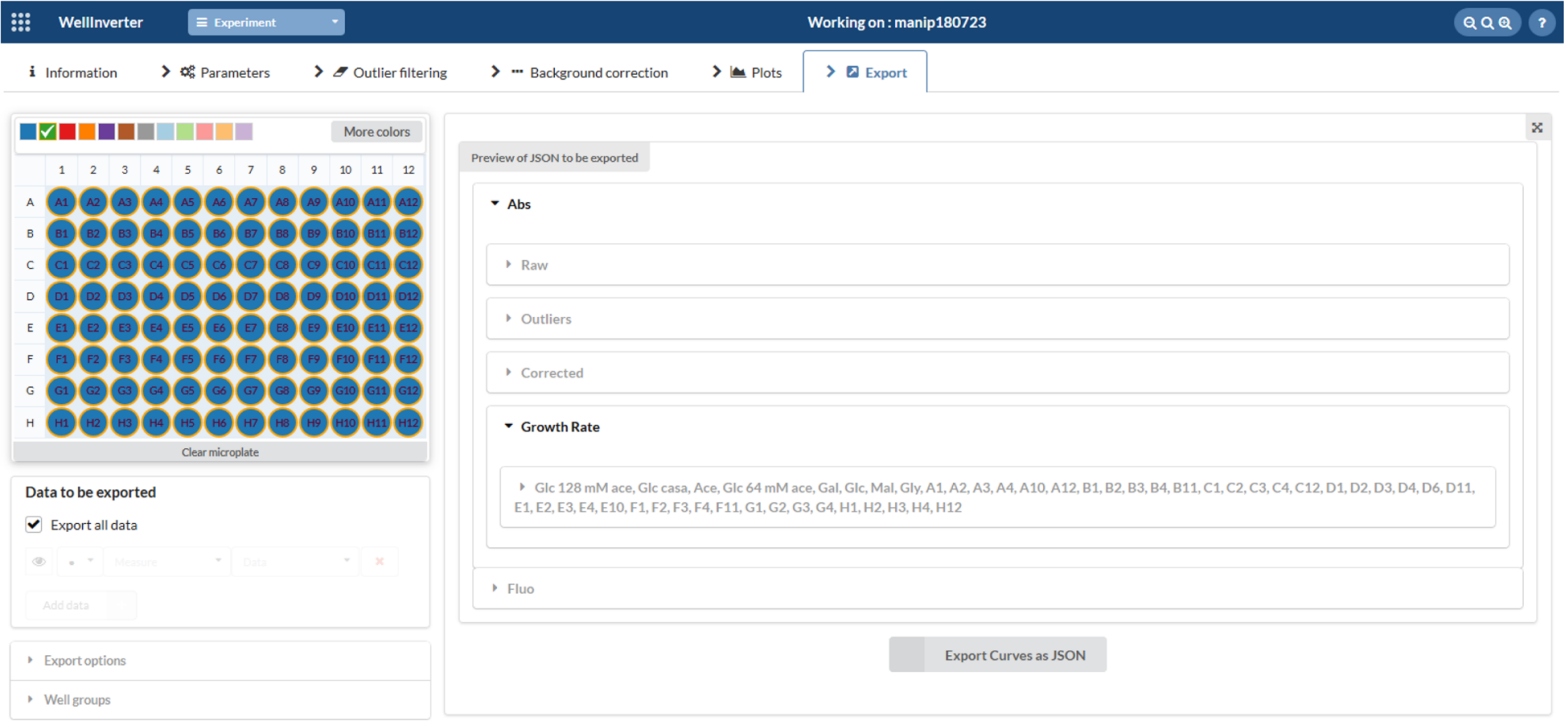
Export of results. Definition of wells and results to be exported to a JSON file for further analysis

While WellInverter allows one to quickly explore the results of an experiment, for many purposes it will be necessary to analyze the data in more detail, to compare and integrate the results over different conditions, *etc.* This will require the use of more flexible, general-purpose scientific software, such as Matlab, R or Python. Moreover, in some cases one may wish to relax the simplifying assumptions that underlie the models presented in the “Estimation algorithms” section. For example, as explained in [22], the models can be generalized so as to distinguish between the transcription and translation processes and to explicitly take into account the maturation of the reporter protein. The WellFARE package contains more sophisticated and time-consuming variants of the estimation algorithms implemented in WellInverter to analyze such extended models. In order to allow further analysis of the data, beyond the functionalities supported by WellInverter, the data and the analysis results can be exported. The *Export* screen allows the user to define the wells and analysis results to export (Fig. 9).

### Growth-rate dependence of the expression of a constitutive gene

**Fig. 10:**
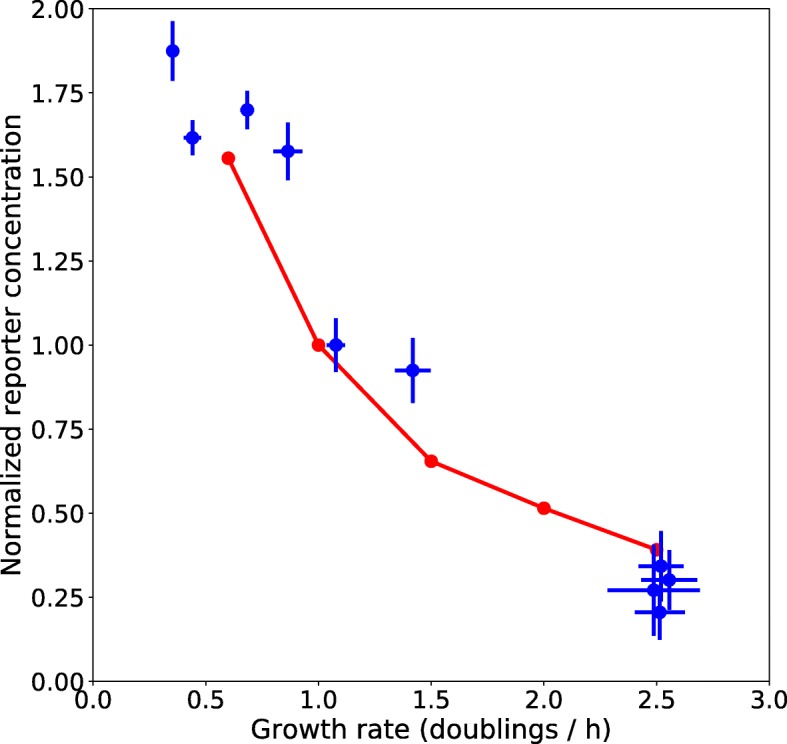
Relation between growth rate and reporter concentration for the pRM promoter. The steady-state growth rate and reporter concentration have been computed for each condition considered (Additional file 2), by taking the mean and standard deviation of the estimates of these quantities over the interval where the cultures are in steady-state exponential growth. The scatter plot shows the mean ± the standard deviation for each condition (blue points and lines, respectively). The growth rates are expressed in doublings per h and the reporter concentrations have been normalized with respect to the estimate for M9 minimal medium with 0.1% glucose. The red dots connected by the red curve are the predicted growth rates and reporter concentrations for a constitutive promoter [28]. The predictions and the data are in very good correspondence, as exemplified by the high *R*^2^ value (0.93). The *R*^2^ value was computed as one minus the ratio of the residual sum of squares divided by the total sum of squares [51]. In order to account for data points below 0.6 doublings/h and above 2.5 doublings/h, we linearly extrapolated the model predictions

The data for the reporter gene experiments have been exported and analyzed separately using Python to determine the relation between the estimated growth rates and reporter protein concentrations. For each medium, we computed the mean and the standard deviation of the steady-state growth rate estimated by WellInverter for (background-corrected) absorbance values between 0.01 and 0.1. In this interval the growth rate of the cultures is observed to be constant and maximal (see Fig. 6 and the discussion in the previous section). Similarly, we computed the mean of the steady-state concentration of the reporter protein produced from the pRM-controlled gene. A scatter plot of pairs of steady-state growth rate and reporter concentration for the different media is shown in Fig. 10 (see Additional file 2 for the data). The growth rates are expressed in terms of the number of doublings per hour, obtained by multiplying the estimated growth rate with 60/ ln2, while the reporter concentrations have been normalized with respect to the value obtained for a reference medium, M9 minimal medium with 0.1% glucose. As can be seen, both the growth rate and the reporter concentration change by a factor of 7-8 over the range of conditions considered here. The uncertainties in the estimated growth rates above 2 doublings per hour are more important due to the higher background levels of the LB medium used in these conditions and the difficulty to maintain steady-state exponential growth over a sufficiently long time-interval in these complex media.

The data can be compared with the theoretical prediction of the reporter concentration for different growth rates in the case that expression of the reporter is controlled by a constitutive promoter [28]. This prediction is obtained from a model of gene expression similar to that being used in WellInverter, using literature data for the different parameters characterizing the expression of a constitutive promoter at different growth rates, such as the transcription and translation rate as well as the gene copy number. The predicted relation between growth rate and reporter concentration corresponds quite well with the observed data for the pRM promoter (*R*^2^=0.93).

This *R*^2^ value should be compared with that obtained for a non-constitutive promoter, p*acs*, which is subject to carbon catabolite repression by the complex Crp-cAMP [42]. The relation between growth rate and reporter concentration for this promoter, determined in exactly the same way as for the pRM promoter using an otherwise identical reporter plasmid [45], is shown in Additional file 3. The correspondence with the model predictions for a constitutive promoter is very weak, as witnessed by the low *R*^2^ value of -0.11, meaning that the model does worse than a baseline model in which it is assumed that the reporter concentration is equal to the observed mean over all growth rates. As a further control, we compared the data for the pRM promoter with the predictions of a model of a promoter repressed by a constitutively expressed transcription factor. For specific parameter values, this model predicts a constant, growth rate-independent reporter concentration [28]. Comparison of this model with the data shows a very poor fit (*R*^2^=0), a result that contrasts with the very good fit of the constitutive promoter model (*R*^2^=0.93).

The above analysis shows that the pRM promoter behaves like a constitutive promoter, in the sense that the growth-rate dependence of the reporter concentration is in good agreement with that expected for a constitutive promoter. This provides another argument, in addition to those listed at the beginning of the “Results” section, for considering this promoter a *bona fide* control when assessing specific regulatory interactions.

## Discussion

Reporter gene experiments using automated microplate readers have become commonplace in microbiology laboratories. However, the interpretation of the data thus generated is far from trivial and quite time-consuming. The difficulties encountered include the size of the data sets and the large number of experimental conditions, the proper substraction of background levels from the fluorescence measurements, and especially the inference of biologically relevant quantities from the primary data. This calls for easily accessible and user-friendly computer tools, based on solid mathematical methods.

The development of mathematical methods for the analysis of reporter gene data has been a lively research area over the past two decades and a variety of methods have been proposed [10, 12–21, 23]. Most of the methods have in common that they are based on an explicit model of the process of gene expression, although they differ in the details of the biochemical processes considered (see, for example, [11, 17, 23] for a discussion of gene expression models used). The models implemented in WellInverter are the simplest possible, reducing gene expression to a single step lumping transcription and translation. While the methods underlying the tool admit multi-step models [22], in our experience one-step models are sufficient in most situations and avoid difficult issues with the parametrization of intermediate transcription, translation, and maturation steps. Moreover, if not sufficient, one-step models at least allow for an initial exploration of the results, before exporting the background-corrected data to tailored analysis scripts for multi-step models.

Most methods for inferring promoter activities reported in the literature are indirect, in the sense that they first empirically smooth the primary fluorescence time-series data, by smoothing splines or other techniques, and then propagate the approximate experimental curves through the gene expression models [10, 17, 23]. In other words, the promoter activities are the output of a computational procedure taking the approximate experimental curves as input. By contrast, direct methods like those used in WellInverter work the other way around; they treat the promoter activity as the input giving rise to an observed output, the fluorescence time-series data [18–20, 22]. This has the conceptual advantage of allowing smoothing to be defined as a regularized data fitting problem on the quantity to be estimated, the promoter activity, and leads to robust results. Moreover, the approach can be easily generalized to the estimation of growth rates and protein concentrations. It should be mentioned though that validation studies with indirect approaches have shown that the latter can also yield excellent results [17, 23]. The simulation study in [23] comparing a direct with an indirect method shows better performance of the latter for data with low time resolution and no bias for low absorbance values. This latter point suggests further work to improve the implementation of regularization in the WellFARE package.

Source code implementing some of the above-mentioned methods is publicly available, but until now few user-friendly computer tools, equipped with a graphical user interface, have been developed to assist biologists in the analysis of reporter gene data. The exceptions that we are aware of, such as WellReader [46] and BasyLICA [19], do not support the same broad scope of functionalities, or are based on proprietary software, or are platform dependent, or a combination of the above. One of the motivations for the development of the original WellInverter software was to make the tool available as a web application, thus avoiding technical issues associated with the cross-platform installation of the software and the supporting libraries. Table 1 summarizes some useful features of available tools.

**Table 1:** Tools for the analysis of fluorescent reporter gene data (names in bold)

Tool	Method	Implementation	Functionalities
**BaSylica** web server [19] ^1^	Kalman filtering	Wamp, mySQL, R	Background correction, computation of promoter activity, graphical user interface
Plate reader package [21, 52] ^2^	Spectral unmixing, Gaussian processes	Python	Background correction, computation of reporter concentration and growth rate
**PromAct** package [23] ^3^	Smoothing splines	R	Computation of promoter activity
**WellInverter** web server [22] ^4^	Linear inversion	JavaScript, Python	Background correction, computation of growth rate, promoter activity, and reporter/protein concentration, graphical user interface
**WellReader** package [46] ^5^	Smoothing splines	Matlab	Background correction, computation of growth rate, promoter activity, and reporter/protein concentration, graphical user interface

^1^ http://genome.jouy.inra.fr/basylica

^2^ http://swainlab.bio.ed.ac.uk/software/platereader/

^3^ https://github.com/soumyakannan/promact

^4^ https://team.inria.fr/ibis/wellinverter/

^5^ https://team.inria.fr/ibis/wellreader-analysis-of-fluorescence-and-luminescence-reporter-gene-data/

Decription of some tools and comparison of key functionalities

The new version of WellInverter presented in this paper improves upon the shortcomings of the original application in a number of important ways. The graphical user interface has been entirely redesigned and rewritten, scalability has been ensured by the development of a parallel computing architecture based on a generic load balancer, and the use of a grammar-based parser generator has made it possible to easily adapt the tool to the import of input files in multiple formats. The new version of WellInverter has been deployed on publicly accessible web servers hosted by the Institut Français de Bioinformatique and Inria, but can also be locally installed (see “Availability and requirements” section).

The capabilities of the tool have been illustrated on the analysis of an *E. coli* dataset. The expression of a green fluorescent reporter protein from a phage promoter has been related to the growth rate, and the resulting curve compared with the predictions from a theoretical model. This has provided further evidence that the phage promoter is constitutive.

The illustrations given above concern fluorescence reporter gene data, but WellInverter is also directly applicable to the analysis of luminescence reporter gene data, since the underlying gene expression models are the same [17]. In the case of luminescence data, however, some additional precautions need to be taken to deconvolve the light signals from a well and its neighbours [47]. More direct experimental quantification of gene products by (absolute) proteomics [48–50] can also be analyzed by means of the tool. In this case, however, one should be aware that rapid changes in gene expression can only be captured if the sampling density is sufficiently high. Whereas the application in this paper concerns bacteria, the gene expression model underlying the methods implemented in WellInverter is general enough to be valid for higher organisms as well.

## Conclusions

We present a novel implementation of WellInverter, a web application for the analysis of fluorescent reporter gene data. We have put in place a parallel computing architecture with a load balancer to distribute the analysis queries over several back-end servers, redesigned the graphical user interface, and developed a plug-in system for the definition of high-level routines for parsing data files produced by microplate readers from different manufacturers. This has resulted in a scalable and user-friendly web service, accessible to a broad audience of biologists and bioinformaticians. The practical applicability of the tool has been illustrated by means of the analysis of the expression pattern of a fluorescent reporter gene transcribed from a constitutive phage promoter.

## Availability and requirements

Access to WellInverter can be obtained in several different ways.

First, for researchers who wish to explore WellInverter before deciding on its use, a demo version can be accessed at the following address:

https://wellinverter.inrialpes.fr

The demo version does not allow the user to upload his or her own data sets. However, sample data sets are supplied: two primary data sets of *E. coli* fluorescent reporter gene experiments as well as a completely processed version of one of these data sets.

Second, WellInverter is accessible through the cloud of the French Institute for Bioinformatics (IFB). The user needs an IFB account that can be created at the following adress:

https://biosphere.france-bioinformatique.fr/cloudweb/login/

Analyzed data cannot be stored on the server and the user needs to download and locally save the results before the end of the session.

Third, WellInverter has been deployed on an Inria server at the following address:

http://ibis-public.inrialpes.fr:8000/

Contrary to the IFB version, no user registration is needed.

Fourth, a stand-alone version of WellInverter is available. The use of this version requires the application to be locally installed, contrary to the options above which only require an Internet connection and a web browser. In the case of intensive use of WellInverter, or data protection issues, this option may be preferable though. Moreover, the analysis results can be locally stored.**Project name:** WellInverter **Project home page:**https://team.inria.fr/ibis/wellinverter/**Operating system(s):** Windows, Linux, and MacOS **Programming language:** JavaScript **Other requirements:** Java (version > 7) **License:** proprietary licence, free for academics **Any restrictions to use by non-academics:** licence needed

The Python library WellFARE, implementing the linear inversion methods on which WellInverter is based, is separately available:

**Project name:** WellFARE **Project home page:**https://github.com/ibis-inria/wellfare**Operating system(s):** Windows, Linux, and MacOS **Programming language:** Python **Other requirements:** NumPy package of Python **License:** LGPL **Any restrictions to use by non-academics:** none

The same holds for the general-purpose load balancer pyLoadBalancer:**Project name:** pyLoadBalancer **Project home page:**https://github.com/ibis-inria/pyLoadBalancer**Operating system(s):** Windows, Linux, and MacOS **Programming language:** Python **Other requirements:** none **License:** GPL **Any restrictions to use by non-academics:** none

## Additional files

Additional file 1 Experimental data..zip archive containing.json file with experimental data that can be uploaded into WellInverter as well as.doc file with plate layout (ZIP 1191 kb)

Additional file 2 Table with data on relation between growth rate and reporter concentration..xlsx file with data used for plotting Fig. 10 in main text and the supplementary figure in Additional file 3 (XLSX 15 kb)

Additional file 3 Figure showing relation between growth rate and reporter concentration for the p*acs* promoter..pdf file (PDF 106 kb)

## Data Availability

The data generated or analysed during this study are included in the Additional files 1 and 2.

## References

[1] Chudakov DM, Matz MV, Lukyanov S, Lukyanov KA (2010). Fluorescent proteins and their applications in imaging living cells and tissues. Physiol Rev.

[2] Giepmans BN, Adams SR, Ellisman MH, Tsien RY (2006). The fluorescent toolbox for assessing protein location and function. Science.

[3] Zaslaver A, Bren A, Ronen M, Itzkovitz S, Kikoin I (2006). A comprehensive library of fluorescent transcriptional reporters for *Escherichia coli*. Nat Methods.

[4] Cox J, Mann M (2011). Quantitative, high-resolution proteomics for data-driven systems biology. Annu Rev Biochem.

[5] Wang Z, Gerstein M, Snyder M (2009). RNA-Seq: a revolutionary tool for transcriptomics. Nat Rev Genet.

[6] QIAGEN. CLC Main Workbench. QIAGEN. https://www.qiagenbioinformatics.com/products/clc-main-workbench/. Accessed 10 Aug 2018.

[7] Berthoumieux S, de Jong H, Baptist G, Pinel C, Ranquet C, Ropers D, Geiselmann J (2013). Shared control of gene expression in bacteria by transcription factors and global physiology of the cell. Mol Syst Biol.

[8] Gerosa L, Kochanowski K, Heinemann M, Sauer U (2013). Dissecting specific and global transcriptional regulation of bacterial gene expression. Mol Syst Biol.

[9] Keren L, Zackay O, Lotan-Pompan M, Barenholz U, Dekel E (2013). Promoters maintain their relative activity levels under different growth conditions. Mol Syst Biol.

[10] Ronen M, Rosenberg R, Shraiman BI, Alon U (2002). Assigning numbers to the arrows: Parameterizing a gene regulation network by using accurate expression kinetics. Proc Natl Acad Sci USA.

[11] Stefan D, Pinel C, Pinhal S, Cinquemani E, Geiselmann J, de Jong H (2015). Inference of quantitative models of bacterial promoters from time-series reporter gene data. PLoS Comput Biol.

[12] Subramanian S, Srienc F (1996). Predictive and interpretive simulation of green fluorescent protein expression in reporter bacteria. J Bacteriol.

[13] Leveau JH, Lindow SE (2001). Predictive and interpretive simulation of green fluorescent protein expression in reporter bacteria. J Bacteriol.

[14] Finkenstädt B, Heron EA, Komorowski M, Edwards K, Tang S, Harper CV, Davis JRE, White MRH, Millar AJ, Rand DA (2008). Reconstruction of transcriptional dynamics from gene reporter data using differential equations. Bioinformatics.

[15] Wang X, Errede B, Elston TC (2008). Mathematical analysis and quantification of fluorescent proteins as transcriptional reporters. Biophys J.

[16] Huang Z, Senocak F, Jayaraman A, Hahn J (2008). Integrated modeling and experimental approach for determining transcription factor profiles from fluorescent reporter data. BMC Syst Biol.

[17] de Jong H, Ranquet C, Ropers D, Pinel C, Geiselmann J (2010). Experimental and computational validation of models of fluorescent and luminescent reporter genes in bacteria. BMC Syst Biol.

[18] Porreca R, Cinquemani E, Lygeros J, Ferrari-Trecate G (2010). Structural identification of unate-like genetic network models from time-lapse protein concentration measurements. Proc. 49th IEEE Conference on Decision and Control (CDC 2010).

[19] Aïchaoui L, Jules M, Le Chat L, Aymerich S, Fromion V, Goelzer A (2012). BasyLiCA: a tool for automatic processing of a Bacterial Live Cell Array. Bioinformatics.

[20] Bansal L, Chu Y, Laird C, Hahn J (2012). Determining transcription factor profiles from fluorescent reporter systems involving regularization of inverse problems. Proc. 2012 American Control Conference (ACC 2012).

[21] Lichten CA, White R, Clark IB, Swain PS (2014). Unmixing of fluorescence spectra to resolve quantitative time-series measurements of gene expression in plate readers. BMC Biotechnol.

[22] Zulkower V, Page M, Ropers D, Geiselmann J, de Jong H (2015). Robust reconstruction of gene expression profiles from reporter gene data using linear inversion. Bioinformatics.

[23] Kannan S, Sams T, Maury J, Workman CT (2018). Reconstructing dynamic promoter activity profiles from reporter gene data. ACS Synth Biol.

[24] Bertero M (1989). Linear inverse and ill-posed problems. Adv Electron Phys.

[25] de Nicolao G, Sparacino G, Cobelli C (1997). Nonparametric input estimation in physiological systems: Problems, methods, and case studies. Automatica.

[26] Wahba G (1990). Spline Models for Observational Data.

[27] Liang S, Bipatnath M, Xu Y, Chen S, Dennis P, Ehrenberg M, Bremer H (1999). Activities of constitutive promoters in *Escherichia coli*. J Mol Biol.

[28] Klumpp S, Zhang Z, Hwa T (2009). Growth rate-dependent global effects on gene expression in bacteria. Cell.

[29] Oppenheim AB, Kobiler O, Stavans J, Court DL, Adhya S (2005). Switches in bacteriophage lambda development. Annu Rev Genet.

[30] Hansen PC (1992). Analysis of discrete ill-posed problems by means of the L-curve. SIAM Rev.

[31] Golub GH, Heath M, Wahba G (1979). Generalized cross-validation as a method for choosing a good Ridge parameter. Technometrics.

[32] Larrabee KL, Phillips JO, Williams GJ, Larrabee AR (1980). The relative rates of protein synthesis and degradation in a growing culture of *Escherichia coli*. J Biol Chem.

[33] Crockford D. The application/json media type for JavaScript Object Notation (JSON). 2006. http://tools.ietf.org/html/rfc4627. Accessed 6 Apr 2019.

[34] Garrett JJ. Ajax: A new approach to web applications. 2006. http://www.adaptivepath.com/ideas/ajax-new-approach-web-applications/. Accessed 6 Apr 2019.

[35] Jones E, Oliphant T, Peterson P, et al. SciPy: Open source scientific tools for Python. 2001. http://www.scipy.org/. Accessed 6 Apr 2019.

[36] Mihalcescu I, Van-Melle Gateau M, Chelli B, Pinel C, Ravanat JL (2015). Green autofluorescence, a double edged monitoring tool for bacterial growth and activity in micro-plates. Phys Biol.

[37] Aho A, Lam M, Sethi R, Ullman J (2006). Compilers: Principles, Techniques, and Tools.

[38] Parr T. The Definitive ANTLR 4 Reference, 2nd ed.Dallas: The Pragmatic Bookshelf.

[39] Wirth N (1977). What can we do about the unnecessary diversity of notation for syntactic definitions?. Commun ACM.

[40] Baba T, Ara T, Hasegawa M, Takai Y, Okumura Y, Baba M, Datsenko KA, Tomita M, Wanner BL, Mori H (2006). Construction of *Escherichia coli* K-12 in-frame, single-gene knockout mutants: the Keio collection. Mol Syst Biol.

[41] Miller JH (1972). Experiments in Molecular Genetics.

[42] Wolfe AJ (2005). The acetate switch. Microbiol Mol Biol Rev.

[43] Bremer H, Dennis PP, Neidhardt FC, Curtiss III R, Ingraham JL, Lin ECC, Low KB, Magasanik B, Reznikoff WS, Riley M, Schaechter M, Umbarger HE (1996). Modulation of chemical composition and other parameters of the cell by growth rate. *Escherichia Coli* and *Salmonella*: Cellular and Molecular Biology.

[44] Klumpp S, Hwa T (2008). Growth-rate-dependent partitioning of RNA polymerases in bacteria. Proc Natl Acad Sci USA.

[45] Baptist G, Pinel C, Ranquet C, Izard J, Ropers D, de Jong H, Geiselmann J (2013). A genome-wide screen for identifying all regulators of a target gene. Nucleic Acids Res.

[46] Boyer F, Besson B, Baptist G, Izard J, Pinel C, Ropers D, Geiselmann J, de Jong H (2010). WellReader: a MATLAB program for the analysis of fluorescence and luminescence reporter gene data. Bioinformatics.

[47] Mauri M, Vecchioni S, Fritz G. Deconvolution of luminescence cross-talk in high-throughput gene expression profiling. ACS Synth Biol. 2019. In press. 10.1021/acssynbio.9b00032 31095908

[48] Picotti P, Clément-Ziza M, Lam H, Campbell DS, Schmidt A, Deutsch EW, Röst H, Sun Z, Rinner O, Reiter L, Shen Q, Michaelson JJ, Frei A, Alberti S, Kusebauch U, Wollscheid B, Moritz RL, Beyer A, Aebersold R (2013). A complete mass-spectrometric map of the yeast proteome applied to quantitative trait analysis. Nature.

[49] Trauchessec M, Jaquinod M, Bonvalot A, Brun V, Bruley C (2014). Mass spectrometry-based workflow for accurate quantification of *E coli* enzymes: how proteomics can play a key role in metabolic engineering. Mol Cell Proteome.

[50] Schmidt A, Kochanowski K, Vedelaar S, Ahrné E, Volkmer B, Callipo L, Knoops K, Bauer M, Aebersold R, Heinemann M (2016). The quantitative and condition-dependent *Escherichia coli* proteome. Nat Biotechnol.

[51] Hamilton LC (1992). Regression with Graphics : A Second Course in Applied Statistics.

[52] Swain PS, Stevenson K, Leary A, Montano-Gutierrez LF, Clark IBN, Vogel J, Pilizota T (2016). Inferring time derivatives including cell growth rates using Gaussian processes. Nat Commun.

