# Supplementary material for: WellInverter: a web application for the analysis of fluorescent reporter gene data
Source: BMC Bioinformatics. 2019 Jun 11;20:309. doi: 10.1186/s12859-019-2920-4 (PMC6558888; doi:10.1186/s12859-019-2920-4)
Supplement: Supplementary file 3 — Figure showing relation between growth rate and reporter concentration for the pacs promoter..pdf file (PDF 106 kb) [file 12859_2019_2920_MOESM3_ESM.pdf]

Additional File 3: Figure showing the relation between growth rate and reporter concentration for the p*acs* promoter*

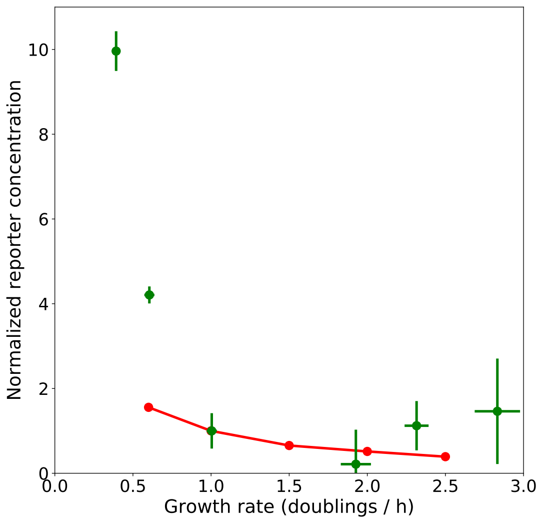

The steady-state growth rate and reporter concentration have been computed for each condition considered (Additional file S2), by taking the mean and standard deviation of the estimates of these quantities over the interval where the cultures are in steady-state exponential growth. The scatter plot shows the mean ± the standard deviation for each condition (green points and lines, respectively). The growth rates are expressed in doublings per h and the reporter concentrations have been normalized with respect to the estimate for M9 minimal medium with

*Supplementary information to 'WellInverter: A web application for the analysis of fluorescent reporter gene data' by Martin *et al.*

0.1% glucose. The red dots connected by the red curve are the predicted growth rates and reporter concentrations for a constitutive promoter. The correspondence between the model predictions and the data are poor, as exemplified by the computed R^2 value (-0.11). The R^2 value was computed as one minus the ratio of the residual sum of squares divided by the total sum of squares. In order to account for data points below 0.6 doublings/h and above 2.5 doublings/h, we linearly extrapolated the model predictions. The negative R^2 value indicates that a baseline model assuming that the reporter concentration equals the observed mean over all growth rates explains the data better than the constitutive promoter model.
