# Supplementary material for: WellInverter: a web application for the analysis of fluorescent reporter gene data
Source: BMC Bioinformatics. 2019 Jun 11;20:309. doi: 10.1186/s12859-019-2920-4 (PMC6558888; doi:10.1186/s12859-019-2920-4)
Supplement: Supplementary file 1 — Experimental data..zip archive containing.json file with experimental data that can be uploaded into WellInverter as well as.doc file with plate layout (ZIP 1191 kb) [file 12859_2019_2920_MOESM1_ESM.zip › plateLayout.doc]

# Plate layout

The measurements are in 3-8 replicates for each condition. M9+glc 0.1%, M9+glc 0.1%+casa 0.1%, M9+gly 0.1%, M9+gal 0.1%, M9+ace 0.1%, M9+mal 0.1%, M9+glc 0.1%+ace 64 mM, M9+glc 0.1%+ace 128 mM, The plate lay-out is as follows:

|  | 1 | 2 | 3 | 4 | 5 | 6 | 7 | 8 | 9 | 10 | 11 | 12 |
| --- | --- | --- | --- | --- | --- | --- | --- | --- | --- | --- | --- | --- |
| A | WT | WT | WT | M9 | WT  pRM-*gfp* | WT  pRM-*gfp* | WT  pRM-*gfp* | WT  pRM-*gfp* | WT  pRM-*gfp* | WT  pRM-*gfp* | WT  pRM-*gfp* | WT  pRM-*gfp* |
| B | WT | WT | WT | M9 | WT  pRM-*gfp* | WT  pRM-*gfp* | WT  pRM-*gfp* | WT  pRM-*gfp* | WT  pRM-*gfp* | WT  pRM-*gfp* | WT  pRM-*gfp* | WT  pRM-*gfp* |
| C | WT | WT | WT | M9 | WT  pRM-*gfp* | WT  pRM-*gfp* | WT  pRM-*gfp* | WT  pRM-*gfp* | WT  pRM-*gfp* | WT  pRM-*gfp* | WT  pRM-*gfp* | WT  pRM-*gfp* |
| D | WT | WT | WT | M9 | WT  pRM-*gfp* | WT  pRM-*gfp* | WT  pRM-*gfp* | WT  pRM-*gfp* | WT  pRM-*gfp* | WT  pRM-*gfp* | WT  pRM-*gfp* | WT  pRM-*gfp* |
| E | WT | WT | WT | M9 | WT  pRM-*gfp* | WT  pRM-*gfp* | WT  pRM-*gfp* | WT  pRM-*gfp* | WT  pRM-*gfp* | WT  pRM-*gfp* | WT  pRM-*gfp* | WT  pRM-*gfp* |
| F | WT | WT | WT | M9 | WT  pRM-*gfp* | WT  pRM-*gfp* | WT  pRM-*gfp* | WT  pRM-*gfp* | WT  pRM-*gfp* | WT  pRM-*gfp* | WT  pRM-*gfp* | WT  pRM-*gfp* |
| G | WT | WT | WT | M9 | WT  pRM-*gfp* | WT  pRM-*gfp* | WT  pRM-*gfp* | WT  pRM-*gfp* | WT  pRM-*gfp* | WT  pRM-*gfp* | WT  pRM-*gfp* | WT  pRM-*gfp* |
| H | WT | WT | WT | M9 | WT  pRM-*gfp* | WT  pRM-*gfp* | WT  pRM-*gfp* | WT  pRM-*gfp* | WT  pRM-*gfp* | WT  pRM-*gfp* | WT  pRM-*gfp* | WT  pRM-*gfp* |
